# Improving risk perception and uptake of voluntary medical male circumcision with peer-education sessions and incentives, in Manicaland, East Zimbabwe: study protocol for a pilot randomised trial

**DOI:** 10.1186/s13063-020-4048-2

**Published:** 2020-01-23

**Authors:** Ranjeeta Thomas, Morten Skovdal, Matteo M. Galizzi, Robin Schaefer, Louisa Moorhouse, Constance Nyamukapa, Rufurwokuda Maswera, Phyllis Mandizvidza, Timothy B. Hallett, Simon Gregson

**Affiliations:** 1 Department of Health Policy, London School of Economics and Political Science, Cowdray House, London, WC2 2AE UK; 2 Section of Health Services Research, Department of Public Health, University of Copenhagen, Øster Farimagsgade 5 opg, B, Postb 15, Building: 15.0.17, 1014 København K, Denmark; 3 Department of Psychological and Behavioural Science, London School of Economics and Political Science, London, WC2 2AE UK; 4 Department of Infectious Disease Epidemiology, Imperial College London, St Mary’s Campus Norfolk Place, London, W2 1PG UK; 5 Biomedical Research and Training Institute, 10 Seagrave, Avondale, Harare, Zimbabwe

**Keywords:** Medical male circumcision, HIV prevention, Incentives, Randomised trial, Zimbabwe

## Abstract

**Background:**

Voluntary medical male circumcision (VMMC) is a key component of combination HIV-prevention programmes. Several high-HIV-prevalence countries in sub-Saharan Africa, including Zimbabwe, are looking to scale up VMMC activities. There is limited evidence on how a combination of social learning from peer education by a role model with different behavioural incentives influences demand for VMMC in such settings.

**Methods/Design:**

This matched-cluster randomised controlled trial with 1740 participants will compare two behavioural incentives against a control with no intervention. In the intervention clusters, participants will participate in an education session delivered by a circumcised young male (“role model”) on the risks of HIV infection and the benefits from medical male circumcision. All participants will receive contributions towards transport costs to access medical male circumcision at participating clinics. Via blocked randomisation, in the intervention clusters participants will be randomly assigned to receive one of two types of incentives – fixed cash payment or lottery payment – both conditional on undergoing surgical VMMC. In two sites, a community-led intervention will also be implemented to address social obstacles and to increase support from peers, families and social structures. Baseline measures of endpoints will be gathered in surveys. Follow-up assessment at 6 months will include self-reported uptake of VMMC triangulated with clinic data.

**Discussion:**

This is the first trial to pilot-test social learning to improve risk perception and self-efficacy and to address the fear of pain associated with VMMC and possible present-biased preferences with front-loaded compensations as well as fixed or lottery-based cash payments. This study will generate important knowledge to inform HIV-prevention policies about the effectiveness of behavioural interventions and incentives, which could be easily scaled-up.

**Trial registration:**

This trial has been registered on ClinicalTrials.gov (identifier: NCT03565588). Registered on 21 June 2018.

## Background

Several studies have demonstrated the effectiveness of male circumcision in reducing HIV incidence [1–3]. Following these findings, voluntary medical male circumcision (VMMC) is now a key component of combination HIV-prevention programmes, especially in high-prevalence regions of sub-Saharan Africa. Several countries have national scale-up programmes to increase male circumcision rates. The VMMC programme in Zimbabwe initially planned to reach 80% coverage among young males (ages 15–29) by 2017. However, uptake was slow and by 2016 only 845,000 VMMCs had been reported [4]. The target date for achieving this goal has since been extended to 2021. Modelling studies have shown that, in order to achieve 80% VMMC coverage by 2018 among 13- to 29-year-old males, 2.17 million VMMCs were required. In addition, 1.30 million other VMMCs would be required between 2018 and 2025 to maintain the 80% coverage [5]. The same study also showed that, in the medium term (2025), prioritising 15- to 24-year-old males would have the greatest impact on HIV incidence. High prevalence of HIV and low male circumcision rates offer great potential for the scale-up of VMMC in Zimbabwe.

However, studies have highlighted several barriers to VMMC uptake. These include fear of pain, reluctance of a relationship partner towards the procedure, cultural barriers and, in the case of working-age men, loss of wages on the days following the procedure because of their inability to work [6]. Behavioural economics offers a further explanation for low uptake of VMMC services based on an individual’s time preferences. If an individual is *present-biased* [7], uptake of VMMC is likely to be low when the individual faces immediate costs (lost wages, pain, and temporary abstinence) and uncertain future benefits (reduced risk of HIV infection). It may be possible to offset such a present bias by means of a peer making information about the future health benefits more salient at the present and of a financial compensation to counterbalance the immediate economic and psychological costs. Behavioural economics has also shown that individuals overweight small-probability events such as winning a lottery [8]. Therefore, the effects of a financial incentive may be greater if, instead of a fixed monetary amount, a lottery-based incentive is offered.

Previous studies offering only financial incentives have found modest effects on VMMC uptake. Fixed incentives for 25- to 49-year-old men showed a 7.4% increase in VMMC uptake within 2 months [9] whereas lottery-based incentives for 21- to 39-year-old men showed no increase within 3 months [10, 11]. Accompanying qualitative studies found that participants reported the fear of pain associated with VMMC as being one of the main reasons individuals did not take up VMMC despite the financial incentive. A recent study [12] found that counselling through “role models” – circumcised young men in a football-focussed intervention – resulted in a 7.6% increase in VMMC uptake amongst 14- to 20-year-old males. This study highlights the role of social learning in enhancing “self-efficacy” [13] for VMMC and explores the possible combination of social learning and behavioural incentives.

The purpose of this study is to evaluate the impact on the uptake of VMMC of an interactive VMMC education session offered by a circumcised “role model”, plus a front-loaded contribution to transport costs for accessing VMMC, along with either (1) a conditional fixed economic incentive compensating for lost wages or (2) a conditional lottery-based economic incentive compensating for lost wages. In all the intervention groups, participants will receive a contribution towards transport costs up-front at the time of randomisation in an attempt to offset the immediate psychological and economic costs. All the financial compensations and lottery prizes will be in the form of cash paid through mobile phone payment systems.

## Methods/Design

### Study hypothesis

The study hypothesises that an VMMC education session offered by a role model – a young male who has previously benefited from VMMC services in this community – addressing the risks of HIV infection, the benefits of VMMC, and the fear of pain associated with VMMC, together with a front-loaded compensation off-setting present-biased preferences, as well as a fixed or lottery-based conditional financial incentive, will improve risk perception and increase uptake of VMMC in HIV-negative young males.

### Study design

**Fig. 1:**
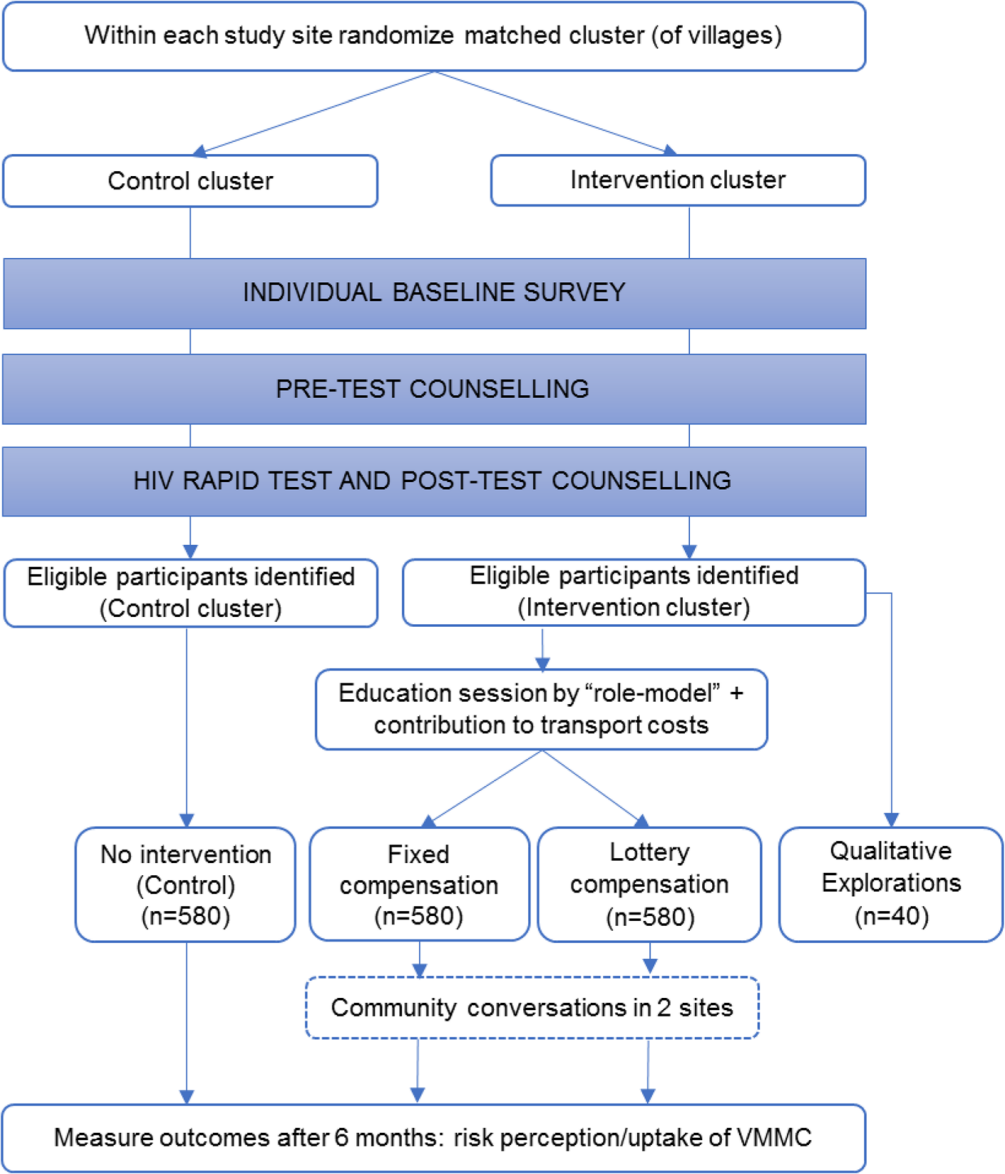
Study design

This is a prospectively registered, three-arm, matched-cluster, randomised controlled trial with single (outcome assessor) masking, intention-to-treat analysis, and 6 months of follow-up (Fig. 1). The study is being implemented in eight sites in Manicaland Province, east Zimbabwe. The study will be implemented site by site. In each study site, a census of all households in the study clusters at baseline will be used to identify 15- to 29-year-old males who are resident within the study site. A detailed individual questionnaire will collect information on primary endpoints and then provider-initiated HIV testing and counselling will be offered. Individuals identified as eligible after this stage will be invited to participate in the intervention.

In each site, two matched clusters of villages will be created. Clusters will be randomly allocated to the intervention arm or to the control arm. Within the intervention clusters, individuals will be further randomly assigned to receive one of two incentives:

A. An education session offered (30 min) by a circumcised male “role model”, an immediate $5 contribution towards transport costs to the health facility, and a $20 payment (about 2 days of wages), conditional on being circumcised within 6 months, or
B. An education session (30 min) offered by a circumcised male “role model”, an immediate $5 contribution towards transport costs, and the following lottery-based financial incentive, conditional on being circumcised within 6 months:

- 5% (1 in 20) chance of receiving $200 (about 10 times 2 days of wages)
- 95% (19 in 20) chance of receiving $10 (about 1 day’s wage)

In addition, in two of the eight sites, a community-engagement intervention using community conversations (CCs) [14, 15] will be implemented to address social and cultural barriers to taking up VMMC and support the above-mentioned individual-level intervention.

Males in the control clusters will receive no intervention over and above the current national standard of care in Zimbabwe. In the study sites, standard of care, which includes some VMMC demand creation activities, comprises information, education and communication (IEC) materials (including posters featuring people such as famous musicians and soccer players who have taken up VMMC) with additional school and community outreach, social marketing, and market-segmentation approaches being tried in some areas. Population Services International functions as a national-level partner of the Zimbabwe Ministry of Health and Child Care (ZMoHCC) in this programme. Until recently, the programme has focussed largely on youth (10–19 years old).

Participant informed consent and data collection will be carried out by trained research assistants. For minors, consent will be sought first from a parent or guardian. For those whose parent or guardian provided consent for study participation, assent will then be obtained from the minors prior to study enrolment. HIV testing will be carried out by appropriately trained and accredited staff following the standard procedures and testing algorithm used in the ZMoHCC routine provider-initiated testing and counselling (PITC) services. HIV field test results will be returned to participants the same day as the survey interview. Participants who test HIV-positive will receive referrals to treatment services as appropriate and according to national guidelines. Participants testing HIV-negative will also receive post-test counselling, and those in the intervention clusters will be invited to participate in the interventions.

Statistical analyses will be carried out by researchers blinded to group allocation. Prior to enrolment, all participants will provide written informed consent. All personal data will be confidential. The study has obtained ethical approvals from the Medical Research Council of Zimbabwe (reference number: MRCZ/A/2243), the Biomedical Research and Training Institute, Zimbabwe Institutional Review Board (reference number: AP140/2017) and the Imperial College London Research Ethics Committee (reference number: 17IC4160).

### Qualitative explorations

Formative qualitative explorations will be embedded into different stages of the study. Formative consultations with local stakeholders and a youth advisory board will provide feedback on the intervention procedures, while qualitative studies will contextualise study outcomes by exploring local norms, experiences and perspectives concerning VMMC. Participants for the qualitative studies will be recruited from settings conveniently chosen because of their rural/urban location and for being subject to both VMMC and CC interventions – allowing a contrast of experiences. Forty young males from the two settings will be invited to participate in either individual interviews (*n* = 12), focus group discussions (*n* = 18) or participatory photography (*n* = 10) with the aim of revealing how they encounter, respond to and negotiate VMMC uptake. The young males will be chosen from the baseline survey by purposeful sampling following the criteria that they have to be between the age of 15 and 29, HIV-negative, sexually active, considered “at risk” (according to a World Health Organization risk screening tool), agreed to be contactable and volunteered to participate. In addition, the young males will be sampled to represent a mix of VMMC intervention experiences to explore their reasons and motivations to participate (or otherwise) in the intervention. Individual interviews with parents of young males (*n* = 8), HIV-prevention service providers (*n* = 12), focus groups with community members (*n* = 6; about 36 participants) and men age 40 and above (*n* = 6; about 36 participants) will also be conducted to gain insight into the broader social context of VMMC and male-friendly HIV-prevention services. Qualitative studies with 40 adolescent girls and young women (AGYW) will also be conducted to provide additional insight into the context that might affect young men’s motivation to take up VMMC. The proposed sample sizes are estimates deemed sufficient to reach theoretical saturation. Transcriptions of the qualitative data will be imported into NVivo 12, a computer-assisted qualitative analysis software, for thematic network analysis [16]. Attention will be paid to the social practices that shape young men’s uptake of VMMC [17] and factors determining progression along the HIV-prevention cascade (HPC) [18].

### Study setting

The study is being conducted in the Manicaland province (in east Zimbabwe), which covers an area of 36,459 km^2^ (14,077 square miles) and has a population of about 1.75 million (2012). Six sites enumerated in a general population cohort survey that ran from 1998 to 2013 [19] – two small towns, two large agricultural estates and two rural villages – will be included in the study, together with two new sites in a high-density urban suburb in Mutare, the provincial capital. The total estimated population size in these areas is 35,760 people. Data available from previous surveys in six study sites included in the current study show that, in 2012–13, HIV prevalences were 13.5% for males aged 15–54 years and 18.8% for females aged 15–54 years.

### Intervention procedures

Research assistants will visit households to recruit study participants. Informed consent will be sought on a one-to-one basis at the respondent’s household or in another private space. Research assistants will describe the study, including its potential risks for the participants and the potential benefits for participants and the local community, and will answer all questions about the study that household members may raise. When an eligible member of a household agrees to participate in the study, he or she will be invited to a central location where the surveys and research activities will take place. The surveys and research activities will take place at health facilities or at nearby venues (e.g. community halls) close to clusters of villages within each study site. The surveys will be administered in a private space on a 1:1 basis by using digital tablets. At the start of the intervention, research assistants will explain the intervention to the participants within the intervention clusters. Participants will be informed that they will be participating in an education session on VMMC, followed by random assignment into one of the two treatment groups. Participants will be told that they will receive incentives if they take up VMMC. In addition, an explanation on the chances of winning the incentive lottery prizes will be provided.

All consenting participants will be included in the education sessions. The sessions will be held in groups of about 10–20 participants. The sessions will be implemented by “messengers” or “role models”, aged 18–30 years, who have undergone VMMC.

During each education session, detailed information on the following will be provided:

- Communication of the risks of HIV infection
- Communication of the effectiveness of VMMC in reducing the risks of HIV infection
- A “personal experience” narration about the role model’s own experience in undergoing and recovering from VMMC, followed by a question-and-answer period

Following the education session, research assistants will:

- Ask each participant to choose a scratch card, which reveals their assignment to an intervention arm. The scratch card will also serve as a VMMC referral card to a participating study clinic where surgical VMMC is available.
- Explain to the participant the referral card and allocated compensation.
- Emphasise that the referral card and compensation are valid for a period of only 6 months.
- Provide information about the participating clinics in the study area where surgical VMMC services will be available free of charge.

**Fig. 2:**
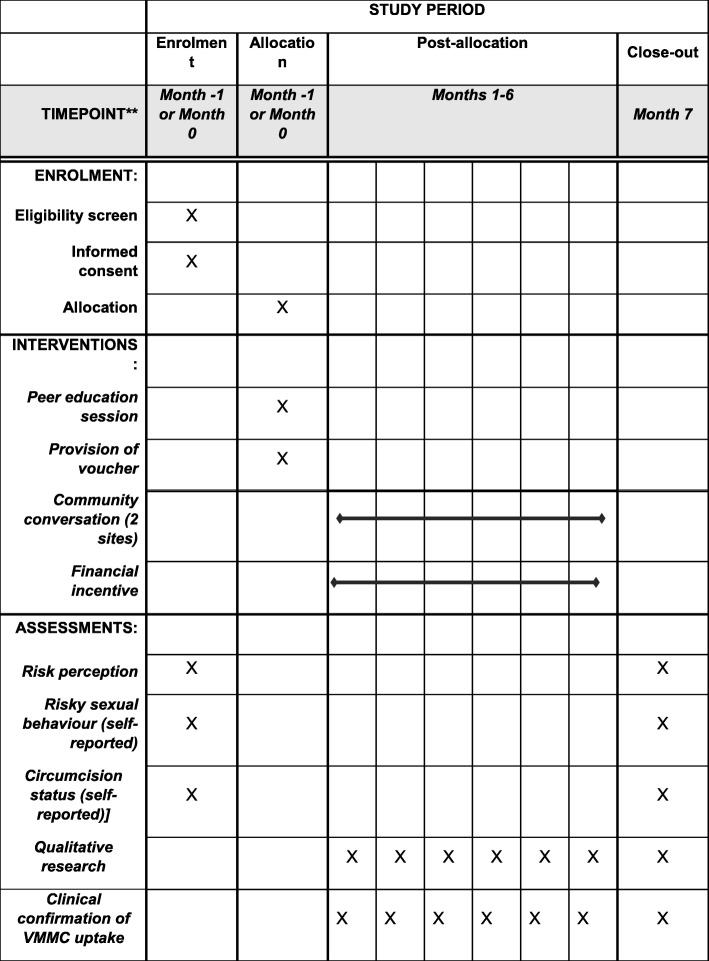
Study schedule of events

Each scratch card will have a unique identification number, which will be matched to their baseline survey information. When participants attend for circumcision and present their referral cards, participating study clinics will retain a section of the referral card. Research assistants will verify that the circumcision procedure was performed by consulting with clinic staff and collect the submitted referral slips. They will then provide participants with the compensation corresponding to their study group via mobile money. This article adhered to the Standard Protocol Items: Recommendations for Interventional Trials (SPIRIT) 2013 checklist (see Additional file 1 and Fig. 2).

#### Further procedures for clusters with community-led interventions

A participatory reflection and action (PRA) approach to community engagement will be introduced to tackle social barriers to uptake of VMMC. The approach of involving local community structures in addressing health challenges is based on strong theoretical foundations [20, 21] and has been applied successfully in other health programmes [22–24] and to tackle gender-based violence in the context of HIV [25]. This approach supports critical thinking and problem solving around key community issues through CCs. CCs involve posing questions and thinking points to formal community groups or informal networks around community challenges and about potential local responses and community resources to tackle these. In Zimbabwe, CCs have helped to raise community HIV competence [26]. This study will be the first to explore whether and how CCs – as a community engagement strategy – can break down community-level barriers with implications for VMMC uptake. In these clusters, the Diocese of Mutare Community Care Programme (DOMCCP) will initiate and support a process to (1) mobilise and work with pre-existing village structures and local community groups that have demonstrated a commitment to fight HIV [27], (2) conduct CCs with formal groups at the village level and generate community action plans, and (3) provide modest funding and technical support to community groups to implement their planned activities. The toolkit has been designed to complement the VMMC intervention by (1) instigating discussions on HIV risk and cementing the salience of HIV-prevention methods and on social preferences and biases to uptake of VMMC, (2) identifying innovative community-led solutions that help AGYW and young males overcome social barriers and re-think risk perceptions, and (3) developing clear steps for communities to prevent the spread of HIV, drawing on local knowledge, resources and existing local activities. Practically, each CC will comprise 10–15 people who, through four meetings, will (1) share their thoughts and experiences of HIV risk and prevention methods, (2) discuss the challenges and factors preventing AGYW and young males from using HIV-prevention methods, (3) explore how to respond, and (4) develop community action plans that correspond to local needs and resources. The participants can be members of any formal group or informal network of people (regardless of age and gender) who at a community level may be able to affect young men’s motivation and access to VMMC services. The facilitation will be carried out by “community conversation facilitators” who have been recruited and trained by the DOMCCP. The facilitators will be local to the communities in which CCs will be implemented and recruited because of their engagement in HIV-prevention work. The CC groups will be supported by the DOMCCP to implement their action plans.

### Inclusion and exclusion criteria

A total of 1740 males will be enrolled on the basis of the following eligibility criteria: (i) if they are between 15 and 29 years of age, (ii) they live in Manicaland province, and (iii) they expect to be resident for the duration of the study. Participants will be excluded if they test HIV-positive at baseline or self-report that they have already been circumcised. To avoid indirect disclosure of the status of HIV-positive individuals, a random sample of 90% of HIV-negative males will be selected to participate in the intervention.

### Randomisation/masking

Within each study site, two matched clusters of villages will be created on the basis of socio-economic characteristics and factors such as distances to trading centres, major roads and health centres. Control and intervention clusters will be constructed to maximise the distance between them to limit potential contamination. Clusters will be randomly allocated to intervention or control. Males in the intervention clusters will receive the intervention. Males in the control clusters will receive no intervention. Males in both clusters will be followed up for measurement of outcomes at 6 months. For the allocation to one of the two treatment groups, blocked randomisation will be used. Blocks of four will be computer-generated and allocated to one of the two treatment arms in a 1:1 ratio. Study staff will be blinded to a block number and the sequence in a block. The treatments will be assigned by using pre-prepared scratch cards that individuals will be asked to select from a set of cards. These cards will also serve as the VMMC referral documentation at participating clinics. Participants will not be blinded from their allocated trial arm due to cluster level randomisation and the nature of the intervention. Study interventions and measurements will occur in separate locations and time points, thus facilitating the blinding of outcome assessors.

### Primary and secondary outcomes

HPCs have been proposed to facilitate (i) identification of gaps in implementation of primary HIV-prevention methods, (ii) understanding of the reasons for these gaps, and (iii) identification and evaluation of promising interventions to address the gaps [28, 29]. Preliminary analysis of HPCs populated using existing study data motivated the selection of some of the outcomes for this study. The analysis found that the low levels of uptake of VMMC in the study population were due largely to low-risk perception for HIV infection – suggesting gaps in motivation [28]. The primary outcome for the study will be the proportion of males taking up VMMC within 6 months measured with self-reports in a follow-up survey conducted after 6 months and confirmed with clinic data. The secondary outcomes will be risk perception – measured in the baseline and follow-up surveys – and self-reported sexual behaviour (e.g. condom use and multiple partners) change measured between the baseline and follow-up surveys.

### Sample size estimates

It is estimated that a sample size of 580 participants per arm will be necessary to detect meaningful minimum effect sizes for the primary endpoint (VMMC uptake). Survey data from the study sites in 2012–13 were used to calculate the mean cluster size, the coefficient of variation in cluster size (0.17 for males), and the intra-cluster correlation coefficient (0.04 using HIV incidence as the outcome). A baseline prevalence estimate for the endpoint indicator (VMMC) was taken to be 30%. It was also assumed that 80% of eligible participants will consent to participate and a further 80% will be followed up at 6 months. The community-led CC intervention will be implemented in the “intervention clusters” in addition to the individual-level VMMC intervention. It will take place in only two cluster pairs, and the analysis will examine the effects of the interventions within these two cluster pairs only so as to allow greater power to detect the effect of the individual-level intervention without CCs. Nevertheless, in the two sites with the combined interventions, the study is powered to detect large impacts on outcome indicators. As a further consequence, there will not be sufficient power to examine the interaction of the effect sizes with and without the addition of the community-level intervention. CCs have been included for exploratory and qualitative reasons, helping us understand the role of social context in shaping uptake of VMMC, for which we consider two sites sufficient.

### Statistical analysis

Analysis of the primary outcome will be carried out on an intention-to-treat basis. For each endpoint, a linear regression will be used to estimate differences in the cluster-level prevalence of the indicator at follow-up. Any indicators unbalanced at baseline will be included as covariates. The Shapiro–Wilk normality test will be used to test for non-normality of the model residuals, and standard non-parametric tests (e.g. Mann–Whitney rank-sum test) will be used in case of non-normality. Effect sizes will be reported with 95% confidence intervals, and results will be considered significant if the *P* value is less than 0.05. Data will be analysed by using STATA.

## Discussion

This trial will evaluate the impact of an education intervention around HIV risks combined with an up-front compensation and conditional incentives to modify behaviour (uptake of VMMC) delivered through fixed or lottery mechanisms in improving perception of HIV risk and uptake of VMMC amongst males aged 15–29 years. The study will provide reliable and high-quality estimates of the average treatment effects since it is a randomised and prospectively registered study. Outcome assessors will be masked, and the analysis will be based on an intention-to-treat approach. Sample size was calculated to provide adequate statistical power to identify possible differences in the study’s primary outcome. In addition, the 6-month follow-up will allow adequate time for participants to take time off work and present at clinics with referral vouchers for VMMC. The 6-month follow-up period is also not too long to measure adjustment in risk perception. Findings from the study will help determine which type of financial incentive is an effective tool to create demand for VMMC and to adjust HIV risk perceptions.

The present study makes several innovative contributions. It combines social learning to improve HIV risk perception and self-efficacy with behavioural incentives and addresses the fear of pain associated with VMMC and possible present-biased preferences with front-loaded compensations as well as with fixed and lottery-based conditional financial incentives. It is thus evaluating the effectiveness of this combination of different prevention interventions in increasing uptake of VMMC in uncircumcised males aged 15–29 years.

This study has limitations. The study does not test the impact of not providing up-front contributions to transport costs or the impact of the education session alone. Another potential limitation is the possibility of lower-than-expected retention rates as males may move away for employment. Any retention challenges will provide valuable information in planning for future research and intervention implementation. Other limitations of this randomised controlled trial could include recruitment challenges and potential sampling bias. Eligibility, recruitment, enrolment and retention will be closely monitored to overcome such challenges.

To achieve the Sustainable Development Goal of ending the AIDS epidemic as a public health threat by 2030, considerable expansions of HIV-prevention efforts are needed. Different demand creation activities must be considered. This study will generate important knowledge to inform HIV-prevention policies about the effectiveness of behavioural incentives which could be easily scaled-up. Given that the costs of the intervention are incurred only once, policy-makers could consider such an intervention, if successful, to be implemented as national campaigns promoted through health facilities at the time of HIV testing or, more generally, as part of health campaigns. In addition, this study will test the usability and acceptability of mobile payments to make payment logistics simpler. These results will be applicable not only to Zimbabwe but also to other settings with high HIV prevalence.

### Trial status

This trial was registered on clinicaltrials.gov (identifier: NCT03565588) on 21 June 2018. https://clinicaltrials.gov/ct2/show/study/NCT03565588. Recruitment commenced on 7 July 2018 and was expected to be completed by 31 December 2019. Follow-up data collection will be completed by 31 August 2020. Protocol version 1.6, 19 April 2019.

## Supplementary information

**Additional file 1.** SPIRIT (Standard Protocol Items: Recommendations for Interventional Trials) 2013 Checklist.

## Data Availability

The datasets generated or analysed (or both) during this study will be made available from the research team on reasonable request. In addition, following completion of the study, the authors plan to make data available by request from the Manicaland Centre for Public Health website (www.manicalandhivproject.org).

## Abbreviations

AGYW: Adolescent girls and young women
CC: Community conversation
DOMCCP: Diocese of Mutare Community Care Programme
HPC: HIV-prevention cascade
VMMC: Voluntary medical male circumcision
ZMoHCC: Zimbabwe Ministry of Health and Child Care

## References

[1] Auvert B, Taljaard D, Lagarde E, Sobngwi-Tambekou J, Sitta R, Puren A (2005). Randomized, Controlled Intervention Trial of Male Circumcision for Reduction of HIV Infection Risk: The ANRS 1265 Trial. PLoS Med.

[2] Gray RH, Kigozi G, Serwadda D, Makumbi F, Watya S, Nalugoda F (2007). Male circumcision for HIV prevention in men in Rakai, Uganda: a randomised trial. Lancet.

[3] Bailey RC, Moses S, Parker CB, Agot K, Maclean I, Krieger JN (2007). Male circumcision for HIV prevention in young men in Kisumu, Kenya: a randomised controlled trial. Lancet.

[4] Zimbabwe Ministry of Health and Child Welfare. Zimbabwe country report: voluntary medical male circumcision (VMMC) bottleneck assessment. Harare; 2013.

[5] Awad SF, Sgaier SK, Ncube G, Xaba S, Mugurungi OM, Mhangara MM (2015). A Reevaluation of the Voluntary Medical Male Circumcision Scale-Up Plan in Zimbabwe. PLoS One.

[6] Brito MO, Caso LM, Balbuena H, Bailey RC (2009). Acceptability of Male Circumcision for the Prevention of HIV/AIDS in the Dominican Republic. PLoS One.

[7] Laibson D (1997). Golden eggs and hyperbolic discounting. Q J Econ.

[8] Tversky A, Kahneman D (1974). Judgment under uncertainty - heuristics and biases. Science..

[9] Thirumurthy H, Masters SH, Rao S, Bronson MA, Lanham M, Omanga E (2014). Effect of Providing Conditional Economic Compensation on Uptake of Voluntary Medical Male Circumcision in Kenya A Randomized Clinical Trial. Jama.

[10] Thirumurthy H, Masters SH, Rao S, Murray K, Prasad R, Zivin JG (2016). The Effects of Providing Fixed Compensation and Lottery-Based Rewards on Uptake of Medical Male Circumcision in Kenya: A Randomized Trial. J Acquir Immune Defic Syndr.

[11] Carrasco MA, Grund JM, Davis SM, Ridzon R, Mattingly M, Wilkinson J (2018). Systematic review of the effect of economic compensation and incentives on uptake of voluntary medical male circumcision among men in sub-Saharan Africa. AIDS Care.

[12] DeCelles J, Hershow RB, Kaufman ZA, Gannett KR, Kombandeya T, Chaibva C (2016). Process Evaluation of a Sport-Based Voluntary Medical Male Circumcision Demand-Creation Intervention in Bulawayo, Zimbabwe. J Acq Imm Def.

[13] Kasprzyk D, Tshimanga M, Hamilton DT, Gorn GJ, Montaño DE (2018). Identification of Key Beliefs Explaining Male Circumcision Motivation Among Adolescent Boys in Zimbabwe: Targets for Behavior Change Communication. AIDS Behav.

[14] Born P (2012). Community conversations: Mobilizing the ideas, skills, and passion of community organizations, governments, businesses, and people: BPS Books.

[15] UNDP (2004). Upscaling Community Conversations in Ethiopia 2004 : unleashing capacities of communities for the HIV/AIDS response.

[16] Attride-Stirling J (2001). Thematic networks: an analytic tool for qualitative research. Qual Res.

[17] Skovdal M (2019). Facilitating engagement with PrEP and other HIV prevention technologies through practice-based combination prevention. J Int AIDS Soc.

[18] Moorhouse L, Schaefer R, Thomas R, Nyamukapa C, Skovdal M, Hallett TB (2019). Application of the HIV prevention cascade to identify, develop and evaluate interventions to improve use of prevention methods: examples from a study in east Zimbabwe. J Int AIDS Soc.

[19] Gregson S, Garnett GP, Nyamukapa CA, Hallett TB, Lewis JJC, Mason PR (2006). HIV decline associated with behaviour change in eastern Zimbabwe. Science.

[20] Prost A, Colbourn T, Seward N, Azad K, Coomarasamy A, Copas A (2013). Women's groups practising participatory learning and action to improve maternal and newborn health in low-resource settings: a systematic review and meta-analysis. Lancet.

[21] Rifkin S, Pridmore P (2001). Partners in planning: information, participation and empowerment.

[22] Lewycka S, Mwansambo C, Rosato M, Kazembe P, Phiri T, Mganga A (2013). Effect of women's groups and volunteer peer counselling on rates of mortality, morbidity, and health behaviours in mothers and children in rural Malawi (MaiMwana): a factorial, cluster-randomised controlled trial. Lancet.

[23] Tripathy P, Nair N, Barnett S, Mahapatra R, Borghi J, Rath S (2010). Effect of a participatory intervention with women's groups on birth outcomes and maternal depression in Jharkhand and Orissa, India: a cluster-randomised controlled trial. Lancet.

[24] Manandhar DS, Osrin D, Shrestha BP, Mesko N, Morrison J, Tumbahangphe KM (2004). Effect of a participatory intervention with women's groups on birth outcomes in Nepal: cluster-randomised controlled trial. Lancet.

[25] Abramsky T, Devries KM, Michau L, Nakuti J, Musuya T, Kyegombe N (2016). The impact of SASA!, a community mobilisation intervention, on women's experiences of intimate partner violence: secondary findings from a cluster randomised trial in Kampala, Uganda. J Epidemiol Community Health.

[26] Campbell C, Nhamo M, Scott K, Madanhire C, Nyamukapa C, Skovdal M (2013). The role of community conversations in facilitating local HIV competence: case study from rural Zimbabwe. BMC Public Health.

[27] Gregson S, Mushati P, Grusin H, Nhamo M, Schumacher C, Skovdal M (2011). Social capital and reduced female vulnerability to HIV infection in rural Zimbabwe. Popul Dev Rev.

[28] Garnett GP, Hallett TB, Takaruza A, Hargreaves J, Rhead R, Warren M (2016). Providing a conceptual framework for HIV prevention cascades and assessing the feasability of empirical measurement with data from east Zimbabwe. Lancet HIV.

[29] Schaefer R, Gregson S, Fearon E, Hensen B, Hallett TB, Hargreaves JR (2019). HIV prevention cascades: a unifying framework to replicate the successes of treatment cascades. Lancet HIV.

